# First-line immunotherapy efficacy in advanced squamous non-small cell lung cancer with PD-L1 expression ≥50%: a network meta-analysis of randomized controlled trials

**DOI:** 10.3389/fonc.2024.1365255

**Published:** 2024-04-24

**Authors:** Wei Chen, Hangmei Liu, Yiwen Li, Wenxin Xue, Shuo Fan, Jingbo Sun, Shui Liu, Yang Liu, Lili Zhang

**Affiliations:** Department of Pharmacy, Emergency General Hospital, Beijing, China

**Keywords:** squamous non-small cell lung cancer, chemotherapy, immune checkpoint inhibitors, network meta-analysis, randomized trials

## Abstract

**Objective:**

The optimal first-line immunotherapy regimen for patients with PD-L1 expression ≥50% in squamous non-small cell lung cancer (Sq-NSCLC) remains uncertain. This study utilized net-work meta-analysis (NMA) to indirectly compare the efficacy of various first-line immuno-therapy regimens in this patient subset.

**Methods:**

Systematic searches were conducted across PubMed, the Cochrane Library, Web of Science, and Embase databases for randomized controlled trials reporting overall survival (OS) and progression-free survival (PFS) outcomes. The search spanned from database inception to November 3, 2023. Bayesian network meta-analysis was employed for a comprehen-sive analysis. To ensure scientific rigor and transparency, this study is registered in the Interna-tional Prospective Register of Systematic Reviews (PROSPERO) under the registration number CRD42022349712.

**Results:**

The NMA encompassed 9 randomized controlled trials (RCTs), involving 2170 patients and investigating 9 distinct immunotherapy regimens. For OS, the combination of camrelizumab and chemotherapy demonstrated the highest probability (36.68%) of efficacy, fol-lowed by cemiplimab (33.86%) and atezolizumab plus chemotherapy (23.87%). Regarding PFS, the camrelizumab and chemotherapy combination had the highest probability (39.70%) of efficacy, followed by pembrolizumab (22.88%) and pembrolizumab plus chemotherapy (17.69%). Compared to chemotherapy, first-line treatment with immune checkpoint inhibitors (ICIs) in Sq-NSCLC pa-tients exhibited significant improvements in OS (HR 0.59, 95% CI 0.47-0.75) and PFS (HR 0.44, 95% CI 0.37-0.52).

**Conclusion:**

This study suggests that, for Sq-NSCLC patients with PD-L1 expression ≥50%, the first-line immunotherapy regimen of camrelizumab plus chemotherapy provides superior OS and PFS outcomes. Furthermore, ICIs demonstrate enhanced efficacy compared to chemotherapy in this patient population.

**Systematic review registration:**

https://www.crd.york.ac.uk/prospero/, identifier: CRD 42022349712.

## 1 Introduction

Globally, lung cancer presents a notable incidence and mortality, with approximately 2.2 million new cases and 1.8 million deaths reported annually, which raises significant public health concerns (1, 2). Non-small cell lung cancer (NSCLC), the most prevalent subtype, accounts for 80%-85% of all lung cancer cases (2).The diagnosis of a majority of NSCLC cases occurs at an advanced stage, leading to a dismal prognosis, characterized by a less than 5% five-year survival rate (3). Some patients suffer from locally advanced or metastatic NSCLC, making surgical resection impractical. Standard treatments for such cases often involve platinum-based paclitaxel chemotherapy and/or radiation therapy (4). However, despite the implementation of these therapeutic strategies, a significant proportion of patients fail to achieve favorable outcomes (5).

In the recent past, the advent of immune checkpoint inhibitors (ICIs) has extended the survival period of NSCLC patients with manageable adverse effects (6, 7). ICIs encompass inhibitors targeting programmed death-1 (PD-1), programmed death ligand-1 (PD-L1), and cytotoxic T-lymphocyte-associated antigen 4 (CTLA-4) (8). Compared to ICIs, chemotherapy may more readily induce drug resistance in patients, potentially contributing to the superior efficacy observed with ICIs (9). This disparity in efficacy might be partly attributed to the differing mechanisms of action between the two treatment modalities. ICIs modulate the immune system by blocking the PD-1/PD-L1 pathway, thereby enhancing immune response (10). This immunomodulation may lead to a more sustained anti-cancer response, reducing adaptive resistance of tumor cells to the treatment (10). In contrast, chemotherapy primarily achieves therapeutic effects by directly destroying cancer cells, but its nonspecific nature might make patients more prone to developing drug resistance (8, 11). NSCLC can be pathologically categorized into squamous non-small cell lung cancer (Sq-NSCLC) and non-squamous cell lung cancer (NS-NSCLC). Patients with these subtypes demonstrate disparities in smoking history, tumor location, and clinical outcomes (12). Therefore, personalized treatment based on the distinct characteristics of each cancer subtype is imperative. In contrast to NS-NSCLC, Sq-NSCLC poses greater therapeutic challenges. Diagnosis often coincides with the presence of comorbidities such as chronic obstructive pulmonary disease and cardiovascular conditions, intensifying the complexity of treatment (13–15). PD-L1 has emerged as a potential prognostic factor and biomarker for predicting which patients are more likely to respond to immunotherapy in NSCLC, thereby refining the target population for potential benefits (16–18). Approximately 30% of advanced NSCLC patients exhibit positive PD-L1 expression (PD-L1 expression ≥50%) as detected by immunohistochemistry (IHC) testing (19).

Presently, for stage IV NSCLC without driver gene mutations and with PD-L1 expression ≥50%, pembrolizumab stands as the preferred first-line treatment option (20). Additionally, due to the lack of randomized controlled trials (RCTs) directly comparing various immune checkpoint inhibitors, selecting the optimal immunotherapeutic approach remains challenging. Nevertheless, ambiguity remains regarding the optimal initial treatment for squamous NSCLC exhibiting PD-L1 expression ≥50%. To address this uncertainty, our study employs network meta-analysis (NMA) to compare the efficacy of various first-line immunotherapy approaches, aiming to identify the most effective treatment regimen. This research endeavors to furnish evidence-based guidance for clinical drug selection and offer improved treatment options for patients with squamous NSCLC.

## 2 Materials and methods

The NMA adhered to the guidelines outlined in the Preferred Reporting Items for Systematic Reviews and Meta-Analyses (PRISMA) extension statement (21). Utilizing Bayesian methods, we conducted indirect comparisons of treatment modalities that lacked direct comparisons in clinical trials (22). This approach facilitated the generation of probabilistic predictions for treatment outcomes. For the sake of transparency, reliability, and originality, the study protocol has been prospectively registered in the International Prospective Register of Systematic Reviews (PROSPERO) under the reference number CRD42022349712.

### 2.1 Data sources and search strategy

To identify eligible studies, systematic searches were performed on PubMed, the Cochrane Library, Web of Science, and Embase databases. We utilized both medical subject headings and textwords in the search process. The search encompassed articles available in these databases from inception until November 3, 2023. The search terms included “immune checkpoint inhibitors,” “PD-1 inhibitor,” “PD-L1 inhibitor,” “CTLA-4 inhibitor,” “nivolumab,” “atezolizumab,” “durvalumab,” “tremelimumab,” “pembrolizumab,” “sintilimab,” “tislelizumab,” “camrelizumab,” “ipilimumab,” and “non-small-cell lung cancer.” The detailed search strategy is available in the **Supplementary Document**.

### 2.2 Inclusion and exclusion criteria

This analysis considered randomized controlled trials meeting the following criteria: (1) patients diagnosed with histologically or cytologically confirmed stage IV Sq-NSCLC, (2) the experimental group receiving immune checkpoint inhibitors, (3) availability of overall survival (OS) or progression-free survival (PFS) data for patients with squamous NSCLC and PD-L1 expression ≥50%, and (4) inclusion of randomized controlled trials specifically investigating first-line treatment regimens.

This study excluded the following: (1) editorials, observational studies, meta-analyse, and reviews, and (2) randomized controlled trials involving the same patient cohort.

### 2.3 Data extraction

Following the PRISMA guidelines, a meticulous process of data extraction was applied to the chosen RCTs. For precision and comprehensiveness, four researchers (W.C, H.L,Y.L. and W.X) autonomously extracted pertinent data, resolving any disparities through discussions with the fifth author(L.Z). The extracted information encompassed details such as the trial name, first author, publication year, trial phase, number of treatment lines, clinical trial identification number, sample size, age and gender distribution of patients, as well as comprehensive particulars about the experimental and control groups. Additionally, clinical outcomes such as OS and PFS were extracted, including the Hazard Ratios (HR) and 95% Confidence Intervals (CIs).

### 2.4 Statistical analysis

Within a Bayesian framework, we employed the “JAGS” and “GeMTC” packages in the R software for NMA (23, 24). This was undertaken to evaluate the effectiveness of different ICIs in the treatment of advanced Sq-NSCLC. A fixed-effects consistency model was utilized, with 25,000 iterations conducted simultaneously across three independent Markov chains. Each chain underwent 60,000 sample iterations. The NMA endpoints included OS and PFS, with effect sizes measured by HR and corresponding 95% CIs. For pairwise meta-analysis, the Revman software was used. Rank probability commands were employed to rank the treatments from best to worst, and differences were considered statistically significant at a two-sided α level < 0.05. One reviewer conducted the statistical analysis, and the results were cross-verified by three additional reviewers to ensure accuracy.

### 2.5 Quality assessment

To guarantee the inclusion of studies adhering to high-quality standards, we utilized the Cochrane Risk of Bias Tool (2.0) for the assessment of randomized controlled trials. This tool scrutinizes the risk of bias across five pivotal domains: the randomization process, potential bias in the implementation of the intended intervention, missing outcome data, measurement of outcomes, and the selection of reported results (25). Following the outcomes of the quality assessment, the included studies were classified as either low risk, high risk, or unclear risk. This categorization ensures the incorporation of only those studies that employ rigorous and reliable methodologies in the NMA.

### 2.6 Sensitivity analysis

Additionally, for optimal alignment with our analysis, model comparison was conducted using the Deviance Information Criterion (DIC). This criterion assesses the relative goodness of fit for fixed-effects and random-effects models, where a lower DIC value signifies superior model fit. Consistency between the fixed-effects and random-effects models is affirmed if the DIC difference is below 5. This method contributed to the selection of the most suitable model for each analysis cohort, ensuring precision in our approach (26).

### 2.7 Heterogeneity analysis

The “anote” command was employed to perform heterogeneity analysis and compute the I^2^ value. Interpretation of I^2^ values is as follows: I^2^ less than 25% signifies low heterogeneity, between 25% and 50% suggests moderate heterogeneity, and exceeding 75% indicates high heterogeneity. For instances of low heterogeneity, a fixed-effects model was applied, while in situations of moderate or high heterogeneity, a random-effects model was employed (27).

## 3 Results

**Figure 1:**
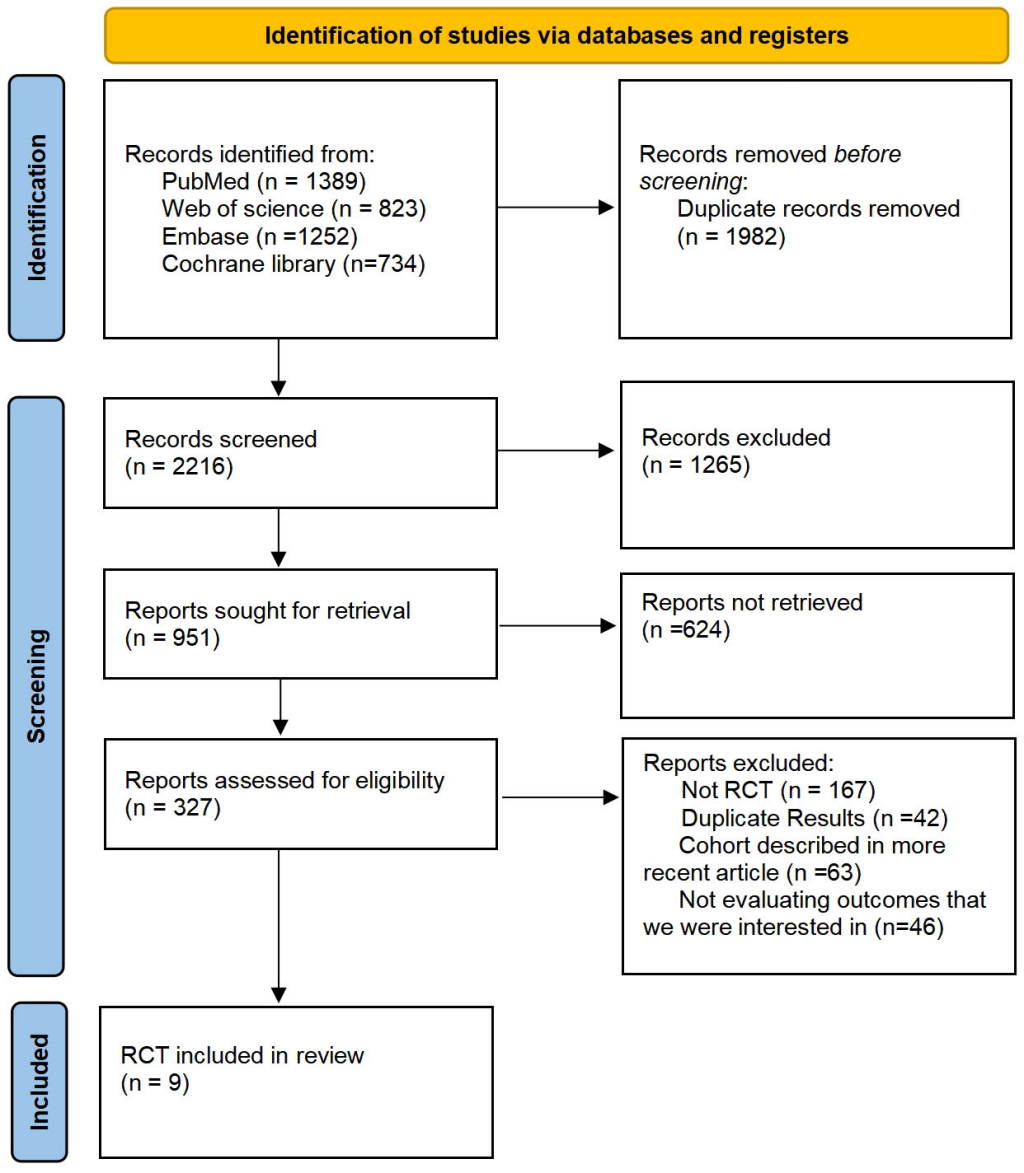
Inclusion of Randomized Controlled Trials: Screening Flowchart.

Following searches in the PubMed, Web of Science, Embase, and Cochrane Library databases, a total of 4198 articles were identified. Post removal of duplicates, 1982 articles were excluded from the analysis. The final selection process is depicted in **Figure 1**, with 9 RCTs being chosen for NMA. This study encompasses 9 RCTs, involving 2170 patients, and assesses 10 treatment regimens for Sq-NSCLC: pembrolizumab (pem), chemotherapy (chemo), pembrolizumab plus chemotherapy (pem-chemo), nivolumab plus ipilimumab (nivo-ipi), atezolizumab plus chemotherapy (atezo-chemo), cemiplimab (cemi), pembrolizumab plus ipilimumab (pem-ipi), camrelizumab plus chemotherapy (camre-chemo), tislelizumab plus chemotherapy (tisle-chemo), and sintilimab plus chemotherapy (sinti-chemo). Detailed results are outlined in **Table 1**.

**Table 1:** Baseline characteristics of the studies incorporated in the network Meta-analysis.

Study	Phase	Design	Line Of treatment	Year	Registered ID	Randomization	Sample Size	Intervention Arm	Control Arm	Reported Outcomes
KEYNOTE-024 (28)	III	open-label	First line	2016	NCT02142738	1:1	29/27	Pembrolizumab 200mg Q3W	Chemotherapy(Platinum-based Chemotherapy Regimens four to six cycles)	OS/PFS
KEYNOTE-407 (29)	III	open-label	First line	2020	NCT02775435	1:1	271/274	Pembrolizumab 200mg Q3W+Chemotherapy(carboplatin and paclitaxel/nab-paclitaxel)	Chemotherapy(carboplatin and paclitaxel/nab-paclitaxel)	OS/PFS
CheckMate-227 (30)	III	open-label	First line	2018	NCT02477826	1:1	94/105	Nivolumab (3 mg per kilogram of body weight every 2 weeks) + Ipilimumab (1 mg per kilogram every 6 weeks)	Platinum doublet chemotherapy	PFS
IMpower-131 (31)	III	open-label	First line	2020	NCT02367794	1:1	343/340	Atezolizumab 1200 mg Q3W +Chemotherapy(Carboplatin AUC 6 Q3W+Nab-paclitaxel 100 mg/m^2^ QW))	Chemotherapy(Carboplatin AUC 6 or Cisplatin 75 mg/m^2^ + Pemetrexed 500 mg/m^2^ Q3W)	OS/PFS
EMPOWER-Lung 1 (32)	III	open-label	First line	2021	NCT03088540	1:1	122/121	Cemiplimab 350 mg Q3W	Platinum-doublet Chemotherapy	OS/PFS
KEYNOTE-598 (33)	III	double-blind	First line	2020	NCT03302234	1:1	77/81	Ipilimumab 1 mg/kg Q6W + Pembrolizumab 200 mg Q3W	Pembrolizumab 200 mg Q3W	OS/PFS
CameL-Sq (34)	III	double-blind	First line	2022	NCT03668496	1:1	37/44	Camrelizumab 200 mg Q3W plus Chemotherapy (carboplatin AUC 5 plus paclitaxel 175 mg/m^2^ Q3W)	Chemotherapy (carboplatin AUC 5 plus paclitaxel 175 mg/m^2^	PFS
RATIONALE 307 (35)	III	open-label	First line	2021	NCT03594747	1:1:1	42/42	Tislelizumab 200 mg plus Chemotherapy (carboplatin AUC 5 plus paclitaxel 175 mg/m^2^ Q3W)	Chemotherapy (carboplatin AUC 5 plus paclitaxel 175 mg/m^2^ Q3W)	PFS
ORIENT-12 (36)	III	double-blind	First line	2021	NCT03629925	1:1	58/63	Sintilimab 200 mg plus Chemotherapy (gemcitabine 1g/m^2^, d1, 8 plus carboplatin AUC 5, d1 or cisplatin 75mg/m^2^ Q3W)	Chemotherapy (gemcitabine 1 g/m^2^, d1, 8 plus carboplatin AUC 5, d 1 or cisplatin 75 mg/m^2^ Q3W)	PFS

### 3.1 Research characteristics

**Figure 2:**
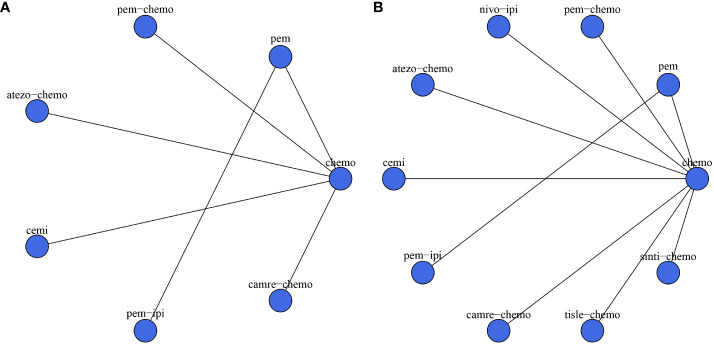
Network diagram. **(A)** overall survival (OS); **(B)** progression-free survival (PFS). In the figure, each point corresponds to a different treatment regimen, and the lines between points represent direct comparisons between two treatments. The thickness of the lines indicates the number of studies for each comparison.

The experimental arms in two RCTs consisted of monotherapy with ICIs (KEYNOTE-024, EMPOWER-Lung 1), while the experimental arms in five other RCTs entailed a combination of ICIs and chemotherapy (KEYNOTE-407, IMpower-131, CameL-Sq, RATIONALE 307, ORIENT-12). Moreover, two additional RCTs featured experimental arms receiving a combination of ICIs (CheckMate-227, KEYNOTE-598). **Figure 2** illustrates a comparative network plot for all observed outcomes. There are a total of 7 relevant first-line treatment regimens with overall survival as the outcome measure, and 10 relevant first-line treatment regimens with progression-free survival as the outcome measure. The network comparison diagram for the various treatment regimens in the nine randomized controlled trials is illustrated in **Figure 2**.

### 3.2 Assessment of included trials

**Figure 3:**
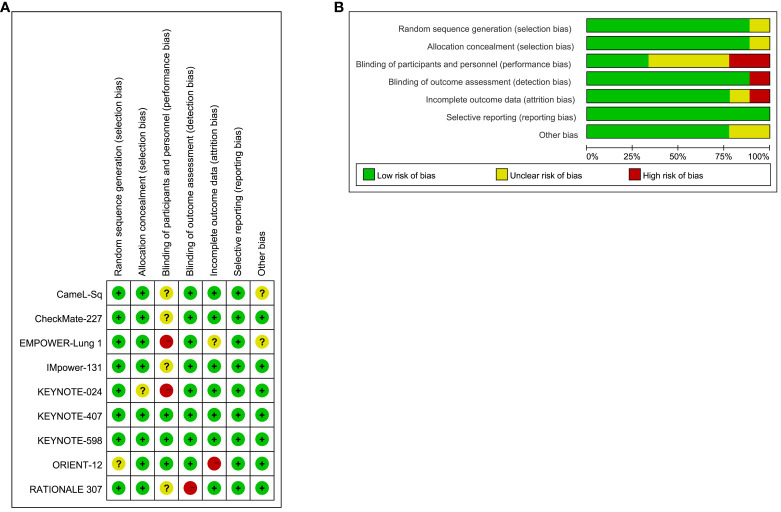
Risk of Bias Figure. **(A)** Summary of risk of bias: The authors rendered judgments for each methodological quality item for every study encompassed in the analysis; **(B)** Graphical representation of methodological quality: The authors’ assessments for each methodological quality item are depicted as percentages across all included studies.

The results of the bias risk assessment for the 9 trials included in the study are illustrated in **Figure 3**. Overall, the bias risk across all studies is considered low, indicating a meticulous scientific approach in the design of these RCTs. Methodological details were validated through a thorough examination of the protocols for each RCT.

Concerning selection bias, eight trials received a low-risk rating, while one trial (ORIENT-12) was classified as unclear. Regarding reporting bias, eight trials were evaluated as low risk, with one trial (KEYNOTE-024) designated as unclear. Implementation bias analysis revealed three trials at low risk, two trials (EMPOWER-Lung 1, KEYNOTE-024) at high risk, and four trials (CameL-Sq, CheckMate-227, IMpower-131, RATIONALE 307) with an unclear rating. Detection bias assessment resulted in eight trials with a low-risk designation, with only one trial (RATIONALE 307) considered high risk. Attrition bias evaluation categorized seven trials as low risk, one trial as high risk (ORIENT-12), and one trial (EMPOWER-Lung 1) as unclear.

All trials received a low-risk rating for reporting bias, primarily due to their analysis based on the intention-to-treat population and the comprehensive reporting of endpoints. However, it is noteworthy that two trials permitted crossover (CameL-Sq, EMPOWER-Lung 1), potentially introducing sources of bias.

### 3.3 Assessment of included trials Pairwise meta-analysis

Through a head-to-head meta-analysis, we evaluated the efficacy of ICIs compared to chemotherapy in patients with Sq-NSCLC), with OS and PFS as the outcome measures.

**Figure 4:**
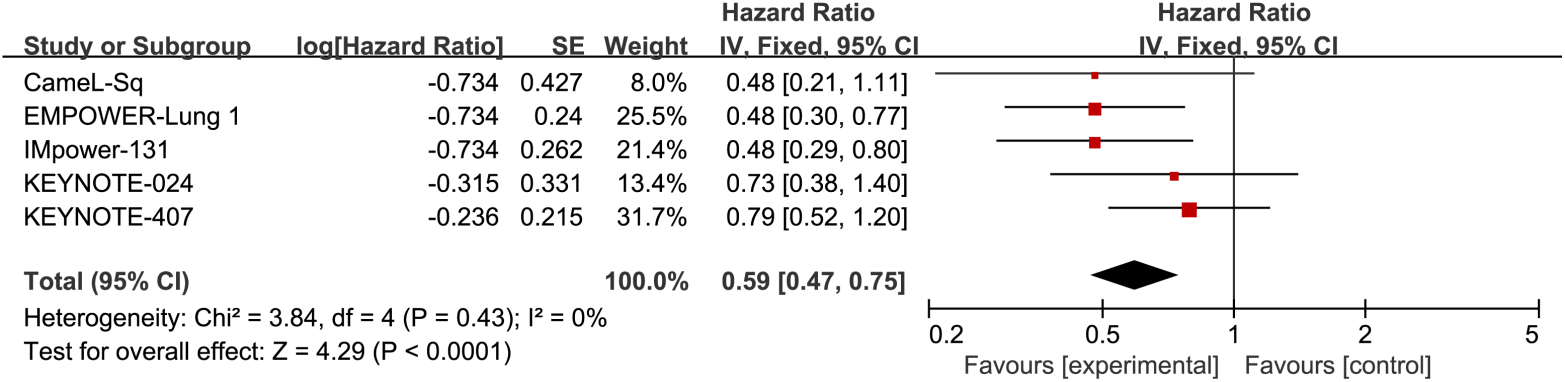
Forest plot illustrating the OS outcome measure. Comparing the Effectiveness of ICIs and Chemotherapy in Advanced Sq-NSCLC among Patients with PD-L1 Expression ≧̸50%.

Five RCTs reported OS, with an I^2^ value of 0, indicating low heterogeneity. Employing a fixed-effects model, the results demonstrated an improvement in OS for S-NSCLC patients treated with ICIs compared to chemotherapy in first-line therapy (HR, 0.59; 95% CI, 0.47-0.75). Refer to **Figure 4** for detailed results.

**Figure 5:**
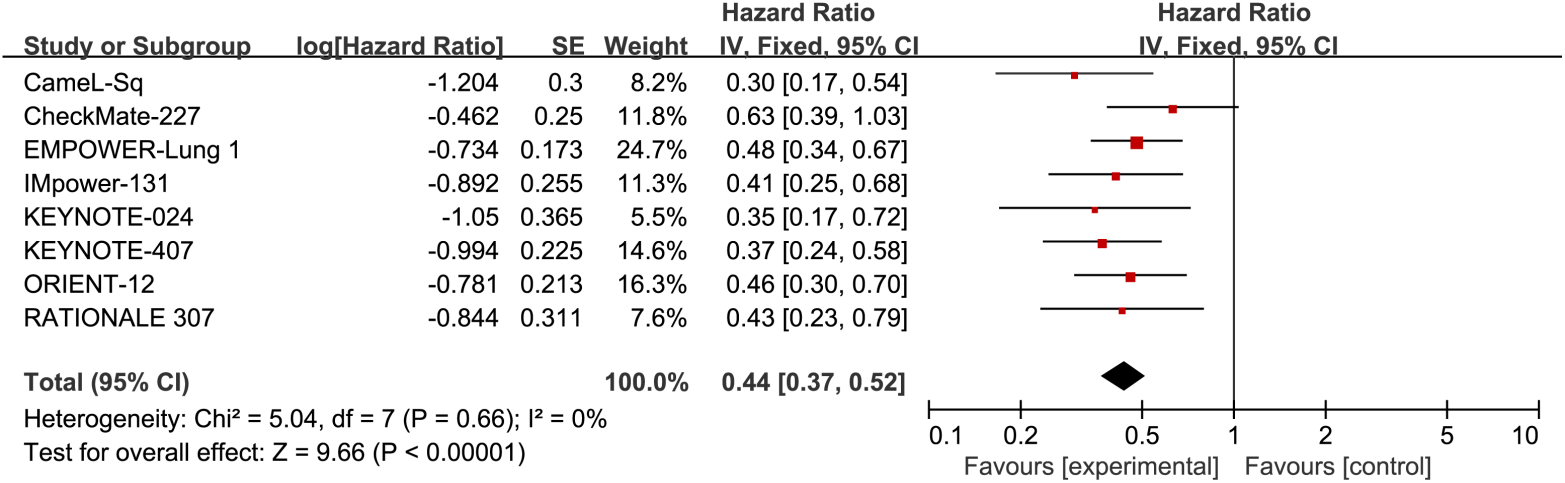
Forest plot illustrating the PFS outcome measure. Comparing the Effectiveness of ICIs and Chemotherapy in Advanced Sq-NSCLC among Patients with PD-L1 Expression ≥50%.

Additionally, eight RCTs reported PFS, with an I^2^ value of 0, suggesting low heterogeneity. The application of a fixed-effects model revealed a noteworthy enhancement in PFS among patients with Sq-NSCLC undergoing ICI treatment in comparison to first-line chemotherapy (HR, 0.44; 95% CI, 0.37-0.52). Specific results can be found in **Figure 5**.

### 3.4 Network meta-analysis

**Figure 6:**
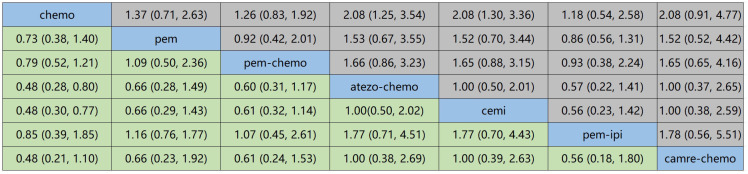
NMA focusing on OS as the primary outcome measure.

**Figure 6** displays the indirect comparison results for OS, demonstrating that Ipilimumab plus Pembrolizumab, compared to chemotherapy, yielded a HR of 0.85 (95% CI, 0.21-1.10).

**Figure 7:**
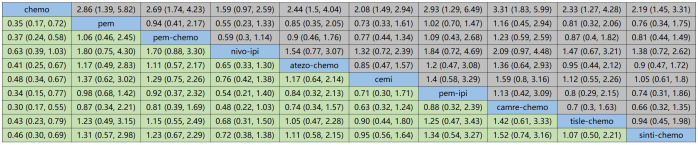
NMA focusing on PFS as the primary outcome measure.

**Figure 7** illustrates the indirect comparison results for progression-free survival (PFS), indicating that the combination of Ipilimumab and Pembrolizumab, as opposed to chemotherapy, yielded a HR of 0.34 (95% CI, 0.15-0.77).

### 3.5 Rankings

**Figure 8:**
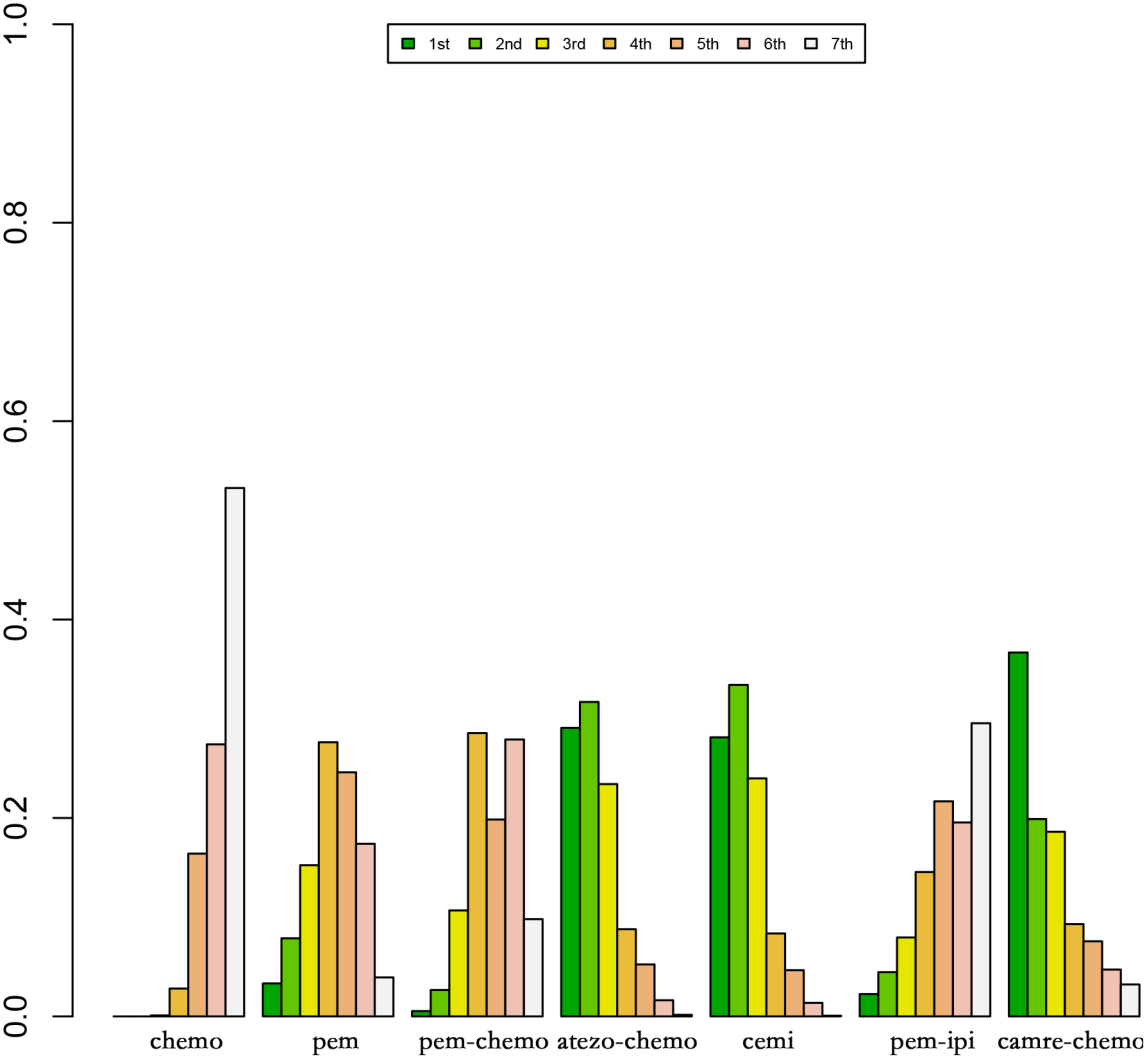
Ranking Plot of OS Treatment Effects. In the first-line treatment, various therapeutic approaches are employed for treating patients with PD-L1 expression ≥50% in Sq-NSCLC.

**Figure 9:**
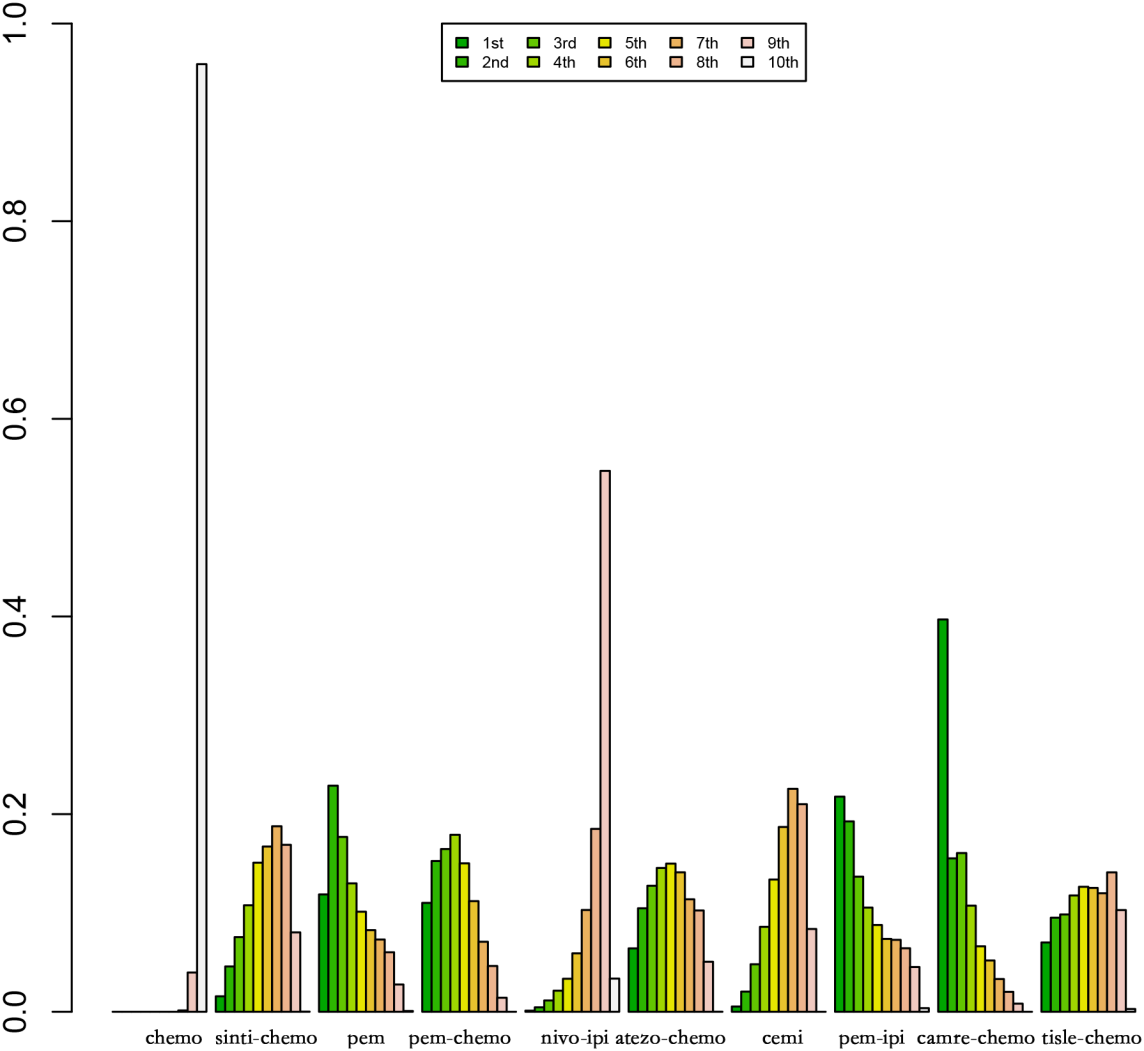
Ranking Plot of PFS Treatment Effects. In the first-line treatment, various therapeutic approaches are employed for treating patients with PD-L1 expression ≥50% in Sq-NSCLC.

**Figure 8** and **Figure 9** represent ranking plots for patients with Sq-NSCLC undergoing various treatment regimens, with OS and PFS as the primary outcome measures.

In OS, the treatment combination of camrelizumab with chemotherapy stands out as the most probable to be the most effective (36.68%), followed by cemiplimab (33.86%) in second place and atezolizumab with chemotherapy (23.87%) ranking third.

Regarding PFS, the most likely most effective treatment is identified as camrelizumab with chemotherapy (39.70%), followed by pembrolizumab (22.88%) in the second position and pembrolizumab with chemotherapy (17.69%) in third place.

## 4 Discussion

This study conducts an extensive NMA to investigate first-line immunotherapy in advanced Sq-NSCLC patients with PD-L1 expression ≥50%. Sq-NSCLC, being more complex compared to NS-NSCLC, is influenced by various factors, including smoking, leading to a higher mutation rate (37). This serves as the justification for concentrating on this specific patient population.

Recognized as a robust predictive biomarker, PD-L1 expression has proven influential, with NSCLC patients showcasing high PD-L1 expression frequently demonstrating a more favorable response to immune checkpoint inhibitors. Therefore, this study explicitly focuses on patients with PD-L1 expression of 50% or higher (38). The goal of this research is to establish a more efficient and personalized first-line immunotherapy strategy for patients with squamous Sq-NSCLC featuring PD-L1 expression of 50% or higher. In the absence of direct head-to-head clinical trials comparing various ICIs, NMA serves as a complementary extension to traditional meta-analysis. NMA utilizes indirect comparisons of interventions from RCTs to rank the efficacy of different ICIs (39).

This study demonstrates that, as a first-line immunotherapy regimen, the combination of camrelizumab and chemotherapy attains optimal values for OS and PFS in patients with advanced Sq-NSCLC exhibiting programmed death-ligand 1 expression of 50% or higher. This is consistent with findings from other NMA that suggest the combination of ICIs and chemotherapy provides the most effective outcomes in the first-line treatment of advanced NSCLC patients with positive PD-L1 expression (40). Pharmacologically, chemotherapy works by directly eliminating cancer cells and inhibiting their proliferation and division, while ICIs block the interaction between PD-1 and PD-L1/PD-L2, thereby inhibiting immune escape (41). These distinct mechanisms, acting synergistically through multiple pathways, enhance treatment effectiveness (41). In a comprehensive NMA study, pembrolizumab emerged as the preferred first-line immunotherapy for advanced NSCLC patients with positive PD-L1 expression, a result that contrasts with the outcomes observed in this study (42). After careful review, it is noted that the published NMA referenced above is from 2021, while the CameL-Sq randomized controlled trial featuring camrelizumab plus chemotherapy was published in 2022. Consequently, the camrelizumab plus Chemotherapy treatment modality was not included in this NMA. It is important to highlight that this NMA primarily focuses on NSCLC patients with PD-L1 expression ≥50%, whereas our study specifically centers on patients with Sq-NSCLC who also exhibit PD-L1 expression ≥50%. In a network meta-analysis, researchers posit that among first-line immunotherapy treatments for NSCLC patients with high PD-L1 expression (≥50%), the use of atezolizumab holds the highest probability of achieving the longest overall survival (OS) (43). Notably, this analysis, published in 2021, does not incorporate the CameL-Sq randomized controlled trial. This underscores the significance of our study, not only contributing to the advancement of personalized treatment approaches but also enhancing the reliability of conclusions by incorporating additional RCTs. Furthermore, this study notes that pembrolizumab, when employed as a first-line treatment for advanced Sq-NSCLC patients with positive PD-L1 expression, exhibits favorable PFS values (HR, 0.35; 95% CI, 0.17–0.72). This study demonstrates that atezolizumab with chemotherapy, when utilized as a first-line treatment for advanced Sq-NSCLC patients with PD-L1-positive expression, exhibits favorable overall survival outcomes (HR, 0.48; 95% CI, 0.28–0.80). In a network meta-analysis evaluating PD-(L)1 inhibitors as monotherapy for first-line treatment in NSCLC patients with high PD-L1 expression, researchers identified cemiplimab as the top-ranking agent for OS, followed by atezolizumab and pembrolizumab (42). In another network meta-analysis assessing immune monotherapy as a first-line treatment for advanced NSCLC patients with PD-L1 expression ≥50%, researchers found that cemiplimab and pembrolizumab exhibited favorable performance in terms of progression-free survival, overall survival, and overall response rate (ORR) (44). In our current study, cemiplimab also demonstrated favorable performance in OS (HR, 0.48; 95% CI, 0.30–0.77). These findings underscore the efficacy of cemiplimab as a monotherapy for the treatment of Sq-NSCLC patients with high PD-L1 expression.

The head-to-head meta-analysis conducted in this study reveals that, for squamous advanced NSCLC patients with PD-L1 expression ≥50%, first-line treatment with ICIs leads to better OS and PFS values compared to chemotherapy, consistent with previous meta-analysis findings (45). This further supports the notion that NSCLC patients with positive PD-L1 expression may experience certain advantages with ICIs over chemotherapy (46).This consistent trend across studies supports the growing understanding of the potential benefits associated with ICIs in this particular patient population, emphasizing the relevance of immunotherapy in the management of advanced NSCLC with elevated PD-L1 expression.

While this study provides valuable insights into personalized treatment strategies for first-line immunotherapy in NSCLC, it is imperative to recognize certain limitations. Firstly, all the RCTs included in this study reported PFS values, but only five reported OS values. This limited availability of OS data may restrict our ability to indirectly compare some treatment regimens based on OS, introducing a potential constraint to our conclusions. Secondly, diverse RCTs in this study utilized different approaches to detect PD-L1 expression in patients. Moreover, in certain trials, researchers did not explicitly specify the method employed to assess PD-L1 expression. The diverse methods for examining PD-L1 expression may lead to misclassification errors. As an example, the utilization of the SP142 assay to detect PD-L1 expression in tumor cells has demonstrated restricted sensitivity, which could potentially affect the precision of the study (47). Thirdly, safety data pertaining to PD-L1 expression of 50% or higher in advanced Sq-NSCLC, which were not documented in the incorporated RCTs, were not subject to assessment in this study. Fourthly, this study utilized OS and PFS values as outcome measures, but it is noteworthy that these may not fully encompass all treatment benefits for patients. When assessing the efficacy of immunotherapy, consideration of other crucial indicators such as quality of life and mental health is warranted. Unfortunately, these aspects are often overlooked by researchers due to the lack of standardized measurement criteria. Finally, due to the limited number of RCTs reporting the efficacy of ICIs in PD-L1 expression ≥50% squamous NSCLC in this study, caution is advised in interpreting the conclusions. This study provides substantial support for advancing personalized treatment strategies, furnishing a wealth of evidence-based guidance for making informed decisions regarding first-line immunotherapy in clinical practice for patients with squamous non-small cell lung cancer. The future direction of development not only includes PD-L1 expression, but also involves identifying predictive biomarkers for treatment response through tumor molecular analysis, such as EGFR mutations in NSCLC (48).

In future research, it is crucial to expand the sample size of RCTs, emphasize standardized PD-L1 expression detection methods, and comprehensively consider a broader array of clinical indicators. Additionally, a comprehensive consideration of a broader array of clinical indicators beyond traditional survival measures is warranted. This inclusive approach will enable a thorough and nuanced evaluation of the application of immunotherapy in patients with squamous NSCLC, shedding light on potential benefits beyond conventional endpoints and fostering a more comprehensive understanding of treatment outcomes.

## 5 Conclusions

This study suggests that in patients with Sq-NSCLC expressing PD-L1 at a level of 50% or higher, the initial immunotherapy selection of camrelizumab in combination with chemotherapy produces superior OS and PFS values. Additionally, for this patient subset, the use of ICIs demonstrates superior efficacy compared to chemotherapy.

## Data availability statement

The original contributions presented in the study are included in the article/**Supplementary Material**. Further inquiries can be directed to the corresponding author.

## Author contributions

WC: Conceptualization, Data curation, Software, Writing – original draft, Writing – review & editing. HL: Conceptualization, Data curation, Supervision, Writing – original draft, Writing – review & editing. YWL: Methodology, Validation, Writing – review & editing. WX: Data curation, Software, Writing – review & editing. SF: Validation, Writing – review & editing. JS: Formal Analysis, Validation, Writing – review & editing. SL: Data curation, Supervision, Writing – review & editing. YL: Visualization, Writing – review & editing. LZ: Supervision, Writing – review & editing.
