# Supplementary material for: First-line immunotherapy efficacy in advanced squamous non-small cell lung cancer with PD-L1 expression ≥50%: a network meta-analysis of randomized controlled trials
Source: Front Oncol. 2024 Apr 24;14:1365255. doi: 10.3389/fonc.2024.1365255 (PMC11080620; doi:10.3389/fonc.2024.1365255)
Supplement: Supplementary file 1 [file DataSheet_1.docx]

Supplementary Material

# **Methods. Supplementary methods**

**Search strategy for Pubmed (MEDLINE/Embase)**：

(((("Carcinoma, Non-Small-Cell Lung"[Mesh]) OR (((((((((((Carcinoma, Non Small Cell Lung[Title/Abstract]) OR (Carcinomas, Non-Small-Cell Lung[Title/Abstract])) OR (Lung Carcinoma, Non-Small-Cell[Title/Abstract])) OR (Lung Carcinomas, Non-Small-Cel[Title/Abstract])) OR (Non-Small-Cell Lung Carcinomas[Title/Abstract])) OR (Non-Small-Cell Lung Carcinoma[Title/Abstract])) OR (Non Small Cell Lung Carcinoma[Title/Abstract])) OR (Carcinoma, Non-Small Cell Lung[Title/Abstract])) OR (Non-Small Cell Lung Carcinoma[Title/Abstract])) OR (Non-Small Cell Lung Cancer[Title/Abstract])) OR (Nonsmall Cell Lung Cancer[Title/Abstract]))) AND (("Lung Neoplasms"[Mesh]) OR (((((((((((((((((Pulmonary Neoplasms[Title/Abstract]) OR (Neoplasms, Lung[Title/Abstract])) OR (Lung Neoplasm[Title/Abstract])) OR (Neoplasm, Lung[Title/Abstract])) OR (Neoplasms, Pulmonary[Title/Abstract])) OR (Neoplasm, Pulmonary[Title/Abstract])) OR (Pulmonary Neoplasm[Title/Abstract])) OR (Lung Cancer[Title/Abstract])) OR (Cancer, Lung[Title/Abstract])) OR (Cancers, Lung[Title/Abstract])) OR (Lung Cancers[Title/Abstract])) OR (Pulmonary Cancer[Title/Abstract])) OR (Cancer, Pulmonary[Title/Abstract])) OR (Cancers, Pulmonary[Title/Abstract])) OR (Pulmonary Cancers[Title/Abstract])) OR (Cancer of the Lung[Title/Abstract])) OR (Cancer of Lung[Title/Abstract])))) AND (((((((((("Immune Checkpoint Inhibitors"[Mesh]) OR (((((((((((((((((((((((((Checkpoint Inhibitors, Immune[Title/Abstract]) OR (Immune Checkpoint Inhibitor[Title/Abstract])) OR (Checkpoint Inhibitor, Immune[Title/Abstract])) OR (Immune Checkpoint Blockers[Title/Abstract])) OR (Checkpoint Blockers, Immune[Title/Abstract])) OR (Immune Checkpoint Blockade[Title/Abstract])) OR (Checkpoint Blockade, Immune[Title/Abstract])) OR (Immune Checkpoint Inhibition[Title/Abstract])) OR (Checkpoint Inhibition, Immune[Title/Abstract])) OR (PD-L1 Inhibitors[Title/Abstract])) OR (PD L1 Inhibitors[Title/Abstract])) OR (PD-L1 Inhibitor[Title/Abstract])) OR (PD L1 Inhibitor[Title/Abstract])) OR (Programmed Death-Ligand 1 Inhibitors[Title/Abstract])) OR (Programmed Death Ligand 1 Inhibitors[Title/Abstract])) OR (PD-1-PD-L1 Blockade[Title/Abstract])) OR (Blockade, PD-1-PD-L1[Title/Abstract])) OR (PD 1 PD L1 Blockade[Title/Abstract])) OR (PD-1 Inhibitors[Title/Abstract])) OR (PD 1 Inhibitors[Title/Abstract])) OR (PD-1 Inhibitor[Title/Abstract])) OR (Inhibitor, PD-1[Title/Abstract])) OR (PD 1 Inhibitor[Title/Abstract])) OR (Programmed Cell Death Protein 1 Inhibitor[Title/Abstract])) OR (Programmed Cell Death Protein 1 Inhibitors[Title/Abstract]))) OR (("pembrolizumab" [Supplementary Concept]) OR ((((SCH-900475[Title/Abstract]) OR (lambrolizumab[Title/Abstract])) OR (MK-3475[Title/Abstract])) OR (Keytruda[Title/Abstract])))) OR (("Nivolumab"[Mesh]) OR ((((((((((Opdivo[Title/Abstract]) OR (ONO-4538[Title/Abstract])) OR (ONO 4538[Title/Abstract])) OR (ONO4538[Title/Abstract])) OR (MDX-1106[Title/Abstract])) OR (MDX 1106[Title/Abstract])) OR (MDX1106[Title/Abstract])) OR (BMS-936558[Title/Abstract])) OR (BMS 936558[Title/Abstract])) OR (BMS936558[Title/Abstract])))) OR (("atezolizumab" [Supplementary Concept]) OR (((((((anti-PDL1[Title/Abstract]) OR (immunoglobulin G1, anti-(human CD antigen CD274) (human monoclonal MDPL3280a heavy chain), disulfide with human monoclonal MDPL3280a kappa-chain, dimer[Title/Abstract])) OR (MPDL3280A[Title/Abstract])) OR (MPDL-3280A[Title/Abstract])) OR (Tecentriq[Title/Abstract])) OR (RG7446[Title/Abstract])) OR (RG-7446[Title/Abstract])))) OR (("durvalumab" [Supplementary Concept]) OR (((MEDI4736[Title/Abstract]) OR (MEDI-4736[Title/Abstract])) OR (Imfinzi[Title/Abstract])))) OR (("avelumab" [Supplementary Concept]) OR (((((MSB-0010682[Title/Abstract]) OR (MSB0010682[Title/Abstract])) OR (bavencio[Title/Abstract])) OR (MSB0010718C[Title/Abstract])) OR (MSB-0010718C[Title/Abstract])))) OR (("camrelizumab" [Supplementary Concept]) OR (((carrelizumab[Title/Abstract]) OR (SHR-1210[Title/Abstract])) OR (SHR 1210[Title/Abstract])))) OR (("tislelizumab" [Supplementary Concept]) OR (BGB-A317[Title/Abstract]))) OR (("sintilimab" [Supplementary Concept]) OR (((IBI 308[Title/Abstract]) OR (IBI308[Title/Abstract])) OR (IBI-308[Title/Abstract]))))) AND (randomized controlled trial[Publication Type] OR randomized[Title/Abstract] OR placebo[Title/Abstract])

**Search strategy for Web of Science**：

"Carcinoma, Non-Small-Cell Lung" OR  "non-small cell lung cancer"   AND  pembrolizumab  OR  nivolumab  OR  atezolizumab  OR  ipilimumab  OR  carboplatin  OR  cisplatin  OR  docetaxel  OR  paclitaxel  OR  "nab paclitaxel"  OR  etoposide  OR  irinotecan  OR  vinblastine  OR  vinorelbine  OR  capecitabine  OR  gemcitabine  OR  bevacizumab  OR  ramucirumab  OR  "programmed cell death 1 receptor"  OR  "pd-l1"  OR  "ctla-4 antigen"

LIMIT-TO: English
